# An outbreak of malaria caused by increase in malaria breeding sites in swamps

**DOI:** 10.11604/pamj.supp.2022.41.1.31258

**Published:** 2022-03-23

**Authors:** Steven Ndugwa Kabwama, Benon Kwesiga, Lilian Bulage, Daniel Kadobera, Alex Riolexus Ario, Julie Rebecca Harris

**Affiliations:** 1 College of Health Sciences, Makerere University School of Public Health, Kampala, Uganda,; 2 Uganda National Institute of Public Health, Kampala, Uganda,; 3 African Field Epidemiology Network, Kampala, Uganda,; 4 Ministry of Health, Kampala, Uganda,; 5 US Centers for Disease Control and Prevention, Kampala, Uganda,; 6 Division of Global Health Protection, Center for Global Health, US Centers for Disease Control and Prevention, Atlanta, United States of America

**Keywords:** Case Study, malaria, outbreak investigation, retrospective cohort, Uganda

## Abstract

On 10^th^ June 2019, routine analysis of malaria surveillance data at the National Malaria Control Division, Ministry of Health in Uganda revealed that there was an unusual increase in the number of malaria cases reported in the Oyam District. On 11^th^ June 2019, the District Health Officer in Oyam District convened a meeting with the District Health Team (DHT) in which the District Biostatistician confirmed that the number of malaria cases had indeed exceeded the upper limit, starting in epidemic week 24 (approximately the week of June 10). The District Health Officer issued a formal request to the Ministry of Health for assistance in dealing with the malaria outbreak in Oyam. Two field epidemiology residents were assigned to work with the District Health Team to investigate the outbreak. The residents followed the steps in conducting vector borne disease outbreak investigations including preparation for field work, establishment of the existence of an outbreak by analyzing surveillance data, descriptive data analysis, hypothesis generation, conducting environmental and entomological assessments, conducting analytic studies with a focus on the utility of retrospective cohort studies as well as reporting findings. This case study teaches trainees in Field Epidemiology and Laboratory Training Programs, public health students, public health workers who are interested or who may participate in vector borne disease outbreak investigation and response.

## How to use this case study

**How to use this case study**: case studies in applied epidemiology allow students to practice applying epidemiologic skills in the classroom to address real-world public health problems. The case studies are used as a vital component of an applied epidemiology curriculum, rather than as stand-alone tools. They are ideally suited to reinforcing principles and skills already covered in a lecture or in background reading. This case study has a Facilitator Guide and a Participant Guide. Each facilitator should review the Facilitator Guide, gain familiarity with the outbreak and investigation on which the case study is based, review the epidemiologic principles being taught, and think of examples in the facilitator´s own experience to further illustrate the points. Ideally, participants receive the case study one part at a time during the case study session. However, if the case study is distributed in whole, participants should be asked not to look ahead.

During the case study session, one or two instructors facilitate the case study for 8 to 20 students in a classroom or conference room. The facilitator should hand out part I and direct a participant to read one paragraph out loud, then progressing around the room and giving each participant a chance to read. Reading out loud and in turns has two advantages. First, all participants engage in the process and overcome any inhibitions by having her/his voice heard. Second, it keeps the all participants progressing through the case study at the same speed. After a participant reads a question, the facilitator will direct participants to answer the question by performing calculations, constructing graphs, or engaging in a discussion of the answer. Sometimes, the facilitator can split the class to play different roles or take different sides in answering the question. As a result, participants learn from each other, not just from the facilitator. After the questions have been answered, the facilitator hands out the next part. At the end of the case study, the facilitator should direct a participant to once again read the objectives on page 1 to review and ensure that the objectives have been met.

**Prerequisites**: for this case study, participants should have received instruction or conducted readings in: Topic 1: Study designs; Topic 2: Prospective and Retrospective Cohort Studies; Topic 3: Odds Ratios and Risk Ratios.

**Target audience:** trainees in the Uganda Field Epidemiology Training Program / Public Health Fellowship Program, other Field Epidemiology and Laboratory Training Programs (FELTPs), public health students, public health workers who may participate in rapid needs assessments and others who are interested in vector-borne disease outbreak investigation and response.

**Level of case study:** intermediate or advanced (intermediate participants should have background in analyzing data from a two-by-two table and in interpreting data from tables).

**Time required**: 3 hours

**Language:** English

## Case study material

- Download the case study student guide (PDF - 687 KB)
- Request the case study facilitator guide
